# Moderating Effect of Mindfulness on the Relationships Between Perceived Stress and Mental Health Outcomes Among Chinese Intensive Care Nurses

**DOI:** 10.3389/fpsyt.2019.00260

**Published:** 2019-04-18

**Authors:** Fang Lu, Yuanyuan Xu, Yongju Yu, Li Peng, Tong Wu, Tao Wang, Botao Liu, Junpeng Xie, Song Xu, Min Li

**Affiliations:** ^1^School of Psychology, Army Medical University, Chongqing, China; ^2^School of Nursing, Army Medical University, Chongqing, China; ^3^Department of Sociology, Sichuan International Studies University, Chongqing, China

**Keywords:** mindfulness, perceived stress, burnout syndrome, depressive symptom, anxiety symptom, subjective well-being, intensive care nurses

## Abstract

This study aimed to explore the potential moderating effect of mindfulness and its facets on the relationships among perceived stress and mental health outcomes (burnout, depression, anxiety, and subjective well-being) among Chinese intensive care nurses. A total of 500 Chinese intensive care nurses completed self-report measures of mindfulness, burnout syndromes, perceived stress, depression, anxiety, and subjective well-being. Correlation and hierarchical multiple regressions were applied for data analysis. Mindfulness moderated the effects of perceived stress on emotional exhaustion (the core component of burnout syndrome), depression, anxiety, positive affect, and negative affect but not on the other two dimensions of burnout and life satisfaction. Further analyses indicated that the ability to act with awareness was particularly crucial in improving the effects of perceived stress on depression. These results further broaden our understanding of the relationships between perceived stress and burnout, depression, anxiety, and subjective well-being by demonstrating that mindfulness may serve as a protective factor that alleviates or eliminates the negative effects of perceived stress on depression, anxiety, burnout syndrome, and subjective well-being and may instigate further research into targeted mindfulness interventions for Chinese intensive care nurses.

## Introduction

The intensive care unit (ICU) is a fast-paced, demanding, and tension-charged environment. Nurses working in the ICU are predisposed to workplace stress owing to direct and indirect exposure to traumatic and critical events, excessive workload (1), high patient care demands (2), long shift work, close contact with death and severe illness (3), and even medical violence (4); however, nurses working in the ICU have limited authority (2). Highly stressful daily events may lead to a heightened stress response combined with negative emotions (5). A growing number of studies have examined mental health among intensive care nursing, but studies on this topic are still rare. Previous studies have produced concordant results demonstrating that ICU nurses show high rates of stress that have resulted in epidemic levels of some work-related mental health problems (6).

The most commonly explored outcome of high-level work-related stress in health care providers is burnout syndrome (7, 8). An increasing number of studies have shown that ICU nurses have an increased prevalence of burnout syndrome (9–11). Burnout is often described as a three-dimensional syndrome that is characterized by emotional exhaustion (EE), depersonalization (DP), and lack of personal accomplishment (PA) (12), which are experienced by 73%, 48%, and 60% of critical care nurses, respectively (10). Compared to nurses in other units, nurses in the ICU treat patients with specific characteristics. Triggers of stress that are different from those experienced in other units include high patient mortality and morbidity, a challenging work environment, and frequent encounters with the critical and traumatic events mentioned above.

The current data show that the prevalence of burnout among critical care staff in different countries has reached alarming levels of approximately 30% in France (11), 16% of nursing staff and 10% of nursing assistants in Spain (13), 56% in a group of pediatric intensive care unit (PICU) staff in Spain (80.9% of nurses and nursing assistants), 84.4% in Argentina (14), and from 25% to 33% in the United States, with up to 86% of the critical care staff experiencing at least one of the three dimensions of burnout. In China, one study indicated that the prevalence of burnout was 83.7% among nurses in the ICU and emergency department (15). Burnout syndrome has been reported to have many negative consequences. It was found that burnout reduces the quality of care (16), lowers patient satisfaction, increases medical error, and leads to interpersonal conflicts, negative emotions, physical symptoms, compulsive behaviors, intention to leave, a higher turnover rate, and absenteeism (9, 10, 12, 17–19).

Perceived stress, depression, and anxiety are strongly and positively related, suggesting a close link between stress and psychopathological symptoms and well-being (20). According to the WHO, over the past two decades, the number of people suffering from anxiety and/or depression worldwide has increased by nearly 50%. Mental illnesses are common among nurses from different countries. Studies have shown that 61.7% of nurses working in the Northeast China Hospital have depressive symptoms (21). The influencing factors and effects of anxiety and/or depression in nurses have been studied. Depressive symptoms among nurses have been linked to stressful work environments, job demand, effort–reward imbalance, etc., which may adversely result in sickness-related absences and affect quality of life and quality of care (21). Well-being is essential for quality of care and productivity. However, prolonged or excessive stress places increasing strain on individuals and has deleterious effects on well-being (22).

Since it is apparent that nursing in the ICU is associated with unique sets of stressors, it is imperative to study the influencing factors and strategies to prevent burnout syndrome, depression, and anxiety, and to promote well-being, and it is crucial for ICU nurses to find ways to manage their stress and prevent the development of these mental health outcomes. According to Folkman et al.’s stress and coping theory (23), stress occurs when one appraises the stressor as exceeding his or her coping resources. The theory suggests that whether perceived stress can predict negative psychological outcomes may depend on the characteristics of the stressors, as well as the individual’s assessment of these stressors and his or her access to coping resources.

Mindfulness may be a potential coping strategy that moderates the negative effects of appraised stress. Mindfulness can be conceptualized as both a trait-like quality (a psychological trait that refers to the tendency to be mindful in everyday life) and a state-like quality (a receptive attention to internal and external experiences) (24). Research of mindfulness interventions in nursing staff has revealed that mindfulness was associated with lower levels of reported stress (25, 26), improved coping with stress and diminished burnout (27–29), decreased EE and anxiety (30), and increased life satisfaction (31). According to Baer et al., mindfulness has five components—observation, description, acting with awareness, nonjudgment of inner experience, and nonreactivity to inner experience (32)—and each facet of mindfulness has specific effects. For example, mindfulness provides individuals the ability to detect the signs of stress by improving awareness (33). Research showed that high dispositional mindfulness may increase awareness of low-level stress symptoms, which, in turn, increases access to coping resources and helps alleviate the negative effects of stress (34). In addition, the ability to observe the effect of the buffer against stress on reduced life satisfaction and depression has been proposed (35). Therefore, it has been suggested that mindful individuals have greater abilities to handle with a set of stressors, which could help moderate the relationship between stress and adverse mental health outcomes (34, 36).

Recently, researchers have shown increasing interest in the stress buffering role of mindfulness. Bergin and Pakenham studied a sample of 481 Australian law school students and found that dispositional mindfulness moderated the effect of perceived stress on anxiety and depression (35). In another student sample, Bodenlos et al. (37) found that the mindfulness facet of nonjudgment acted as a buffer of well-being. By studying 292 patients with gastrointestinal cancer, Zhong et al. (38) concluded that the relationship between psychological symptoms and perceived stress was notable in patients with low dispositional mindfulness. Ciesla et al. (36) conducted a prospective study of 78 high school students and found that nonjudgment and nonreactivity mitigated the effects of daily stress on the daily changes in dysphoric affect. In a sample of 382 Swedish adults, Bränström et al. (34) found that dispositional mindfulness buffered the negative impact of perceived stress on mental health. However, most of the studies mentioned above were carried out in Western societies, and whether mindfulness also constitutes a health resource for ICU nurses working in Chinese societies is insufficiently explored.

Given the high rate of stress in ICU professionals found in previous studies combined with the lack of information focused on both the negative and positive indicators of mental health outcomes, this study pursues two specific goals: first, to test how stress, mindfulness, depressive and anxiety symptoms, and subjective well-being (SWB) are interrelated in Chinese ICU nurses and, second, to detect whether mindfulness moderates the relationship between perceived stress and the related mental health outcomes mentioned previously. Moreover, out of the studies on the moderating role of mindfulness, there have been few studies examining the stress-moderating role of all five mindfulness facets illustrated by Baer et al. (39). Therefore, this study will explore the moderating effects of observation, description, acting with awareness, nonjudgment of inner experience, and nonreactivity to inner experience on perceived stress and other variables. We hypothesized that

1. High levels of mindfulness would be associated with better mental health outcomes for ICU nurses, and mindfulness would moderate the relationship between perceived stress and mental health outcomes.
2. Compared to ICU nurses with lower levels of mindfulness, the association between higher perceived stress and worse mental health outcomes would be weaker and better health outcomes would be more prevalent in nurses with higher levels of mindfulness.

## Materials and Methods

### Participants and Procedure

From March 2016 to April 2017, a total of 500 intensive care nurses were recruited from hospitals in Xinjiang, Ningxia, Beijing, Shandong, Heilongjiang, Fujian, Chongqing, Sichuan, Guizhou, Yunnan, and Hubei provinces through purposive sampling methods. The nurses were recruited from local hospitals, military hospitals, first-class hospitals, and second-senior class hospitals in the northwestern, northern, northeastern, southeastern, southwestern, and central regions of China. The ages of the 500 participants ranged from 20 to 52 years (mean = 27.68 years, SD = 4.275). Among the participants, 41 were male and 459 were female, and 246 were unmarried and 256 were married. None of the participants had meditation experience or practiced meditation before.

The research design of this cross-sectional study was previously approved by the Ethics Committee of Army Medical University. Informed consent forms were obtained from participants who completed questionnaires, including the Five Facet Mindfulness Questionnaire (FFMQ), the Positive and Negative Affect Schedule (PANAS), the Perceived Stress Scale (PSS), the Maslach Burnout Inventory–Human Services Survey (MBI-HSS), the Satisfaction with Life Scale (SWSL), the Center for Epidemiological Studies Depression Scale (CES-D), and the Self-Rating Anxiety Scale (SAS). The uniform instructions were used for the test.

### Measures

*Demographic information.* Participants provided demographic information including age, gender, marital status, and meditation experience.

*Burnout.* The Maslach Burnout Inventory–Human Services Survey (MBI-HSS) was used to assess the burnout syndrome of the human service professionals. The MBI-HSS includes 22 items in three job-related dimensions: emotional exhaustion (EE), depersonalization (DP), and personal accomplishment (PA), and the 22 items were rated on a seven-point Likert scale, ranging from 0 = “never” to 6 = “every day” (40). Samantha Mei-Che Peng from The Hong Kong Polytechnic University translated the Chinese version of the MBI-HSS, which demonstrated good validity and reliability in China (41). There were nine items in the EE dimension, five items in the DP dimension, and eight items in the PA dimension. The Cronbach’s alpha values were 0.86 for EE, 0.76 for DP, and 0.76 for PA. Based on previous studies, medical professionals with scores >26 on the EE subscale, >9 on the DP subscale, or <34 on the PA subscale are defined as having high burnout in that field.

*Mindfulness.* Translated from the FFMQ (39), the Chinese version of the Five Facet Mindfulness Questionnaire (Ch-FFMQ) is a questionnaire that measures dispositional mindfulness with 39 items (42). These items are rated on a five-point Likert scale ranging from 1 (never or very rarely true) to 5 (very often or always true), focusing on five facets of mindfulness: Observation, Description, Acting with awareness, Nonjudgment of inner experience, and Nonreactivity to inner experience (32). The Observation facet evaluates a tendency to observe or focus on external and internal experiences, such as emotions, thoughts, and sensations. Description assesses the propensity to describe and categorize these experiences with words. The Acting with awareness facet measures a sense of bringing full awareness and constant attention to the present experience or activity. The Nonjudging of inner experience facet measures a nonevaluative perspective toward inner experiences and cognition. Nonreactivity to inner experience assesses the tendency to allow feelings and thoughts to surface then leave, without becoming stuck on them or becoming overwhelmed by them. It has been shown that the FFMQ has good internal consistency (39), and the Cronbach’s alpha values for the Ch-FFMQ are acceptable (42).

*Depression.* The Chinese version of the Center for Epidemiological Studies Depression Scale (CES-D) (43) was used to assess depressive symptoms. The CES-D consists of 20 items and is widely used to measure the epidemiology of depressive symptoms, with a focus on emotional components and depression. Participants in this study rated the frequency of occurrence of depressive symptoms on a four-point Likert scale: 0 = “never,” 1 = “sometimes,” 2 = “frequently,” or 3 = “always.” The Chinese version of the CES-D has good reliability and validity (α = 0.89) (44). Individuals with a total CES-D score 16 or higher were defined as having “depressive symptoms” (45), which was further defined as mild for CES-D scores between 16 and 26 or moderate to severe for scores 27 or higher, which was based on the established criteria (46, 47).

*Anxiety.* Zung’s Self-Rating Anxiety Scale (SAS) (48) served as an assessment tool to reflect the severity of anxiety symptoms reported by participants during the 7 days prior to completing the questionnaire. The SAS contains 20 items rated on a four-point Likert scale from 1 = “never” to 4 = “always,” with a total raw score ranging from 20 to 80 and index score ranging from 25–100,respectively. The SAS and the cutoff point defined have been used extensively in studies of anxiety in China, and the Chinese translation of the survey has been previously validated (49). In terms of an investigation of Chinese National Normative Scores, Chinese researchers defined SAS scores >50 as the cutoff point for symptom severity associated with anxiety disorders (50). The score is positively related to the severity of anxiety. Cronbach’s alpha coefficient for the scale was 0.849 in the present study.

*Perceived stress.* The Perceived Stress Scale (PSS) is extensively used to measure the extent to which an individual considers his or her life to be stressful and to assess how controllable and unpredictable the stress has been over the past month (51). This scale has 14 items, with 7 items negatively stated and 7 items positively stated, which are rated on a five-point Likert scale from 1 = “not at all” to 5 = “always.” Higher scores indicated a higher level of perceived stress. The total score for all 14 items ranges from 14 to 70. The Chinese version of the Perceived Stress Scale (CPSS) was developed by Yang and Huang (52) to assess perceived stress with good reliability and validity. The alpha coefficient values for the positive and negative subscales were 0.86 and 0.77, respectively (53).

*Subjective well-being.* Subjective well-being (SWB) is a composite concept that includes a high level of satisfaction with life, more positive emotions, and fewer negative emotions (54). Greater life satisfaction, high positive affect, and low negative affect are often considered as indices of greater SWB (55). Thus, the measure of SWB was shown to be consistent with the Positive Affect and Negative Affect Schedule (PANAS) (56) and with the Satisfaction with Life Scale (SWLS) (57). The PANAS are 20 adjectives that indicate positive affect, such as enthusiasm and activity, and negative affect, such as hatred, contempt, and feelings of guilt. Participants are asked to rate each mood state listed on a five-point Likert scale ranging from 1 = “very lightly or not at all” to 5 = “very much.” The internal consistency coefficients in our sample for the positive and negative affect subscales were 0.86 and 0.83, respectively. The SWLS is a five-item scale used to measure global life satisfaction. Participants were asked to describe the extent to which they agree or disagree with each item on a seven-point Likert scale from 1 = “totally disagree” to 7 = “totally agree.” Higher scores indicate greater perceived life satisfaction. The internal consistency coefficient of SWLS in the present study was 0.89.

### Analyses

Descriptive analyses were based on the mean (M), standard deviation (SD), and range. Preliminary analyses were carried out to test the relationships between demographic variables (age, gender, and marital status), predictors, and outcome variables to identify demographic variables that must be controlled for in further analyses. Through independent-samples *T* test, we found that unmarried ICU nurses perceived more stress and reported higher DP than married ICU nurses. Therefore, age and marital status were controlled for in further analyses.

Hierarchical multiple regression analyses were performed to determine whether stress interacts with mindfulness, as well as the five facets individually, in the prediction of burnout (including three dimensions), depression, anxiety, and SWB (including three components). To increase the interpretability of the moderation model and to control for multicollinearity, all continuous variables were centered, and then the product terms were calculated, which represent the two-way interactions among stress, depression, anxiety, well-being, and mindfulness (58). The variables were entered in the following steps: a) the controlled demographic variables, b) the total perceived stress by ICU nurses, c) the total mindfulness, and d) the interaction term of stress × total mindfulness. Mindfulness was entered after perceived stress to explore the unique differences in mindfulness in the test results. **Table 1** shows a summary of all the separate hierarchical multiple regression models. To explore the potential main and moderate effects of the five mindfulness facets, regression analyses were conducted repeatedly using the five facets to replace total mindfulness at step 3a, and five interactions between each facet and the total perceived stress were input in step 4b. Simple slope analyses were conducted to illustrate the significant interactions among all the moderating models. According to Jaccard et al. (59), the unstandardized regression coefficients (B) of the regression lines for ICU nurses for low (1 SD below mean) and high (1 SD above mean) effects of the moderating variable were adopted. SPSS 19.0 was applied for data analyses.

**Table 1:** Hierarchical multiple regression analyses of perceived stress and mindfulness on burnout, depression, anxiety, and subjective well-being.

Steps and independent variables	Dependent variables											
	Emotional exhaustion			Depersonalization			Personal achievement			Depression		
	β	Total *R* *^2^*	Δ*R* *^2^*	β	Total *R* *^2^*	Δ*R* *^2^*	β	Total *R* *^2^*	Δ*R* *^2^*	β	Total *R* *^2^*	Δ*R* *^2^*
Step 1												
Age	0.05			−0.01			0.08			−0.03		
Marital status	−0.05	0.01	0.01	−0.10	0.01	0.01	0.03	0.01	0.01	0.07	0.01	0.01
Step 2												
Stress	0.42***	0.18	0.17***	0.28***	0.01	0.08***	−0.38***	0.14	0.14***	0.55***	0.31	0.30***
Step 3a												
Total FFMQ	−0.78***	0.64	0.46***	−0.41***	0.21	0.12***	0.02	0.14	0.00	−0.23***	0.34	0.04***
Step 4a												
Stress × Total FFMQ	−0.09**	0.65	0.01**	−0.01	0.21	0.00	−0.01	0.13	0.00	−0.13**	0.36	0.02**
Step 3b												
Observing	−0.04			0.04			−0.00			0.05		
Describing	−0.09			−0.02			0.14**			−0.04		
Awareness	−0.25***			−0.25***			0.09			−0.31***		
Nonjudging	−0.06			0.06			−0.11*			−0.02		
Nonreactivity	−0.08	0.23	0.05***	0.11*	0.17	0.09***	0. 07	0.19	0.05***	−0.07	0.38	0.08***
Step 4b												
Stress × Observing	0.08			0.09			−0.03			0.02		
Stress × Describing	−0.05			−0.03			−0.02			0.03		
Stress × Awareness	0.01			0.02			0.04			−0.14**		
Stress × Nonjudging	0.06			0.07			−0.03			−0.03		
Stress × Nonreactivity	−0.01	0.24	0.01	0.10	0.19	0.02	0.08	0.20	0.01	−0.06	0.40	0.02*
Steps and independent variables	Dependent variables											
	Life satisfaction			Positive affect			Negative affect			Anxiety		
	β	Total *R* *^2^*	Δ*R* *^2^*	β	Total *R* *^2^*	Δ*R* *^2^*	β	Total *R* *^2^*	Δ*R* *^2^*	β	Total *R* *^2^*	Δ*R* *^2^*
Step 1												
Age	0.02			0.06			0.07			0.09		
Marital status	−0.20***	0.05	0.05***	−0.01	0.00	0.00	0.03	0.00	0.00	−0.01	0.01	0.01
Step 2												
Stress	−0.43***	0.22	0.18***	−0.37***	0.14	0.13***	0.49***	0.24	0.23***	0.50***	0.26	0.25***
Step 3a												
Total FFMQ	0.13**	0.24	0.01**	0.16**	0.16	0.00**	−0.25***	0.28	0.05***	−0.65***	0.57	0.32***
Step 4a												
Stress × Total FFMQ	0.01	0.24	0.00	−0.11**	0.16	0.01**	−0.08*	0.29	0.01*	−0.10**	0.58	0.01**
Step 3b												
Observing	0.12*			0.19***			0.02			−0.01		
Describing	0.07			0.15**			−0.03			−0.08		
Awareness	0.02			0.03			−0.25***			−0.22***		
Nonjudging	−0.03			−0.01			−0.00			0.03		
Nonreactivity	0.09	0.27	0.05***	0.12*	0.24	0.10***	−0.04	0.28	0.05***	−0.07	0.30	0.04***
Step 4b												
Stress × Observing	0.02			−0.01			−0.08			0.03		
Stress × Describing	−0.02			−0.01			0.07			0.00		
Stress × Awareness	0.03			−0.07			−0.06			−0.01		
Stress × Nonjudging	−0.02			−0.04			−0.08			0.01		
Stress × Nonreactivity	0.05	0.27	0.00	−0.02	0.25	0.01	−0.06	0.28	0.01	−0.04	0.30	0.00

## Results

### Descriptive Statistics

The descriptive statistics and internal reliability coefficients for each measure are shown in **Table 2**.

**Table 2:** Descriptive statistics and psychometric properties of the major study variables (*n* = 500).

Main study variables	M	SD	Internal reliability α	Range
Total perceived stress (PSS)	40.33	6.10	0.762	19–61
Total mindfulness (FFMQ)	117.98	10.21	0.890	77–160
Observing (O)	21.72	4.68	0.727	8–37
Describing (D)	24.51	4.34	0.768	8–40
Acting with awareness (AWA)	28.42	4.83	0.828	8–40
Nonjudging (NJ)	24.44	4.57	0.780	9–39
Nonreactivity (NR)	18.96	3.19	0.605	10–30
Emotional exhaustion (MBI_EE)	20.87	9.89	0.853	0–53
Depersonalization (MBI_DP)	7.88	5.33	0.803	0–30
Personal achievement (MBI_PA)	26.96	8.46	0.819	3–48
Depressive symptoms (CES-D)	19. 90	6.49	0.945	9–46
Anxiety symptoms (SAS)	46.82	11.20	0.849	25–85
Life satisfaction (SWLS)	19.69	6.23	0.885	5–35
Positive affect (PA)	27.39	6.07	0.873	11–45
Negative affect (NA)	21.12	6.70	0.902	10–50

PSS, Perceived Stress Scale; FFMQ, Five Facet Mindfulness Questionnaire; CES-D, The Center for Epidemiological Studies Depression Scale; SAS, Self-Rating Anxiety Scale; MBI, Maslach Burnout Inventory–Human Service; SWB, PA + SWLS-NA.

According to the cutoff of dimensions of burnout, depression, and anxiety mentioned in measures, the prevalence of these psychological symptoms was common. Of the 500 Chinese ICU nurses, 56% (280/500) were positive for symptoms of depression and 35.2% (176/500) were positive for symptoms of anxiety. There is a prevalence of burnout syndrome, with 84% (420/500) of nurses having positive symptoms in at least one of the three individual dimensions: 23% (115/500) were positive for EE, 27% (135/500) were positive for DP, and 77.8% (389/500) were positive for lack of PA.

**Table 3** confirms that perceived stress was significantly positively associated with EE, DP (two negative dimensions of burnout), depressive and anxiety symptoms, and negative affect and was negatively associated with personal achievements (the positive dimension of burnout) and SWB. On the other hand, total mindfulness was negatively correlated with EE, DP, depression, and anxiety and was positively correlated with personal achievements and SWB (all *p* < 0.01). The mindfulness facets of description and acting with awareness were significantly negatively associated with EE, DP, anxiety, and depression and were significantly positively correlated with personal achievements and SWB. Observation was significantly positively correlated with DP, personal achievement, and depression; nonjudgment was significantly negatively correlated with EE, DP, personal achievement, and depression; and nonreactivity was significantly positively correlated with DP and personal achievement.

**Table 3:** Summary of bivariate correlations among the main study variables (*n* = 500).

Main study variables	1	2	3	4	5	6	7	8	9	10	11	12	13	14	15
1. Total stress	–														
2. Total mindfulness	−0.49**	–													
3. Observing	−0.04	0.17**	–												
4. Describing	−0.46**	0.52**	0.24**	–											
5. Acting with awareness	−0.45**	0.45**	−0.33**	0.29**	–										
6. Non-judging	−0.16**	0.12**	−0.49**	−0.17**	0.34**	–									
7. Non-reactivity	0.01	0.09*	0.45**	0.10*	−0.38**	−0.52**	–								
8. Emotional exhaustion	0.40**	−0.80**	−0.01	−0.29**	−0.35**	−0.10*	0.02	–							
9. Depersonalization	0.26**	−0.48**	0.13**	−0.16**	−0.36**	−0.13**	0.19**	0.61**	–						
10. Personal achievement	−0.37**	0.20**	0.01*	0.32**	0.20**	−0.09*	0.10*	−0.17**	−0.18**	–					
11. Depressive	0.55**	−0.44**	0.01*	−0.30**	−0.49**	−0.16**	0.08	0.45**	0.35**	−0.27**	–				
12. Anxiety	0.48**	−0.74**	−0.02	−0.32**	−0.36**	−0.05	−0.01	0.82**	0.54**	−0.24**	0.55**	–			
13. Subjective well-being	−0.56**	0.80**	0.09	0.41**	0.36**	0.02	0.07	−0.82**	−0.51**	0.29**	−0.53**	−0.82**	–		
14. Life satisfaction	−0.44**	0.30**	0.20**	0.30**	0.14**	−0.08	0.15**	−0.26**	−0.10*	0.27**	−0.33**	−0.28**	0.52**	–	
15. Positive affect	−0.37**	0.30**	0.29**	0.35**	0.09	−0.14**	0.22**	−0.24**	−0.13**	0.39**	−0.20**	−0.25**	0.44**	0.36**	–
16. Negative affect	0.47**	−0.43**	0.06	−0.26**	−0.41**	−0.13**	0.07	0.43**	0.42**	−0.20**	0.54**	0.54**	−0.56**	−0.19**	0.05

*p < 0.05; **p < 0.01.

As hypothesized, after controlling for the demographic variables of age and marital status, the entry of perceived stress in step 2 indicated that perceived stress was positively associated with all negative outcome variables, such as EE, DP, depressive and anxiety symptoms, and negative affect. Moreover, as expected, perceived stress was negatively associated with all positive outcome variables, such as personal achievements, life satisfaction, and positive affect. After the entry of total mindfulness in step 3a, the results revealed a unique variance in all outcomes, except personal achievement, after controlling for perceived stress, age, and marital status. The entry of the five mindfulness facets in step 3b showed a set of significant effects on all outcome variables after controlling for perceived stress, age, and marital status. Observation was positively correlated with life satisfaction and positive affect; description was positively associated with personal achievement and positive affect; awareness was significantly correlated with all negative outcome variables and none of the positive outcome variables; nonjudgment was negatively correlated with personal achievement, which contrasted with our expectation; and nonreactivity had a positive main effect on positive affect and on DP.

**Figure 1:**
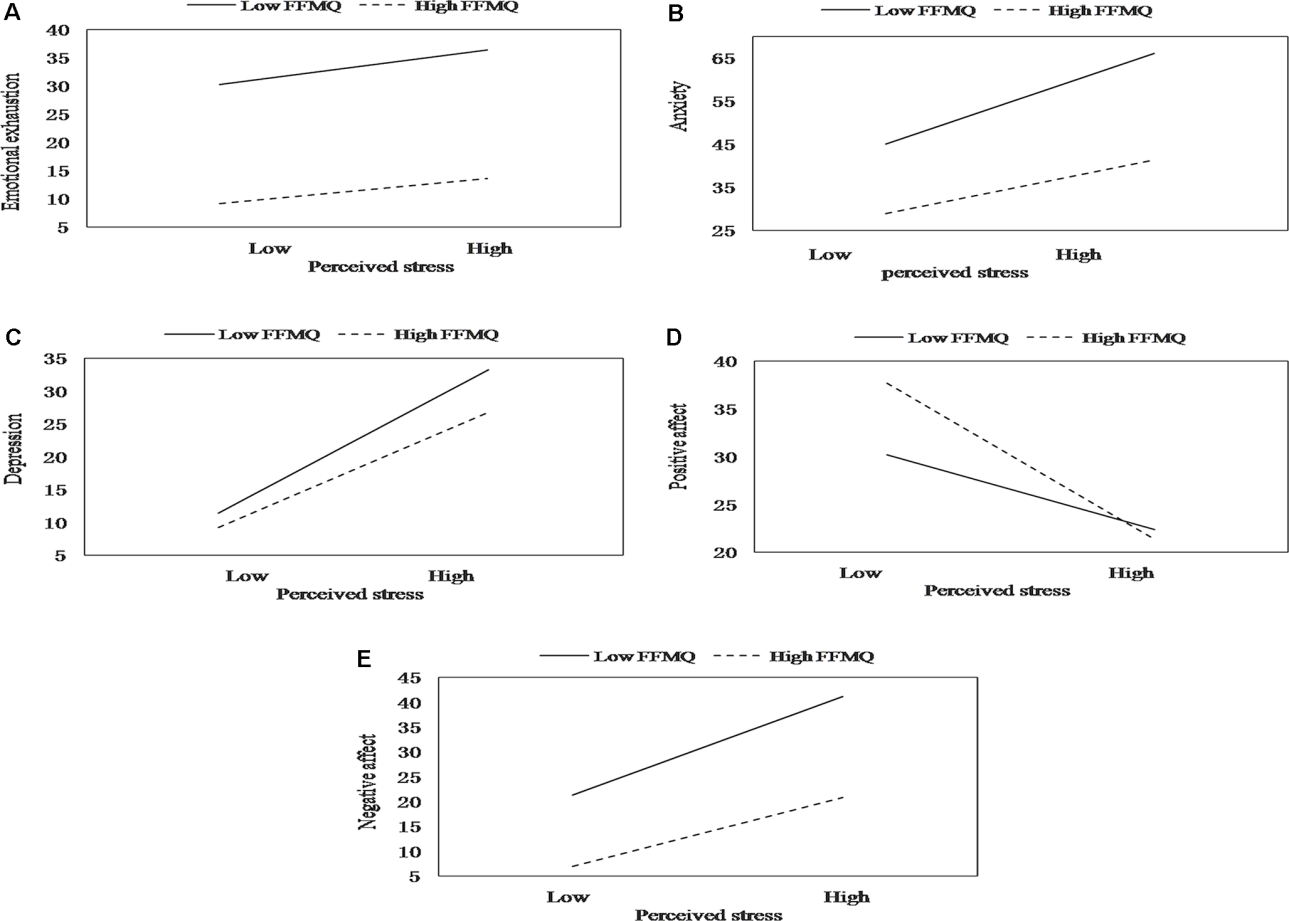
The moderating effect of mindfulness on the relation between stress and emotional exhaustion **(A)**, depression **(B)**, anxiety **(C)**, positive affect **(D)**, and negative affect **(E)**.

The entry of the interaction term stress × total mindfulness in step 4a resulted in significant results related to EE, depression, anxiety, positive affect, and negative affect. This indicated that mindfulness alleviated the effect of perceived stress on EE, depression, anxiety, positive affect, and negative affect. A simple slopes analysis showed that perceived stress predicted EE at both low (*B* = 0.20, *t* = 1.51, *p* < 0.05) and high levels of mindfulness (*B* = 0.15, *t* = 0.57, *p* < 0.05), but the association between perceived stress and EE was weaker when the mindfulness level was high (see **Figure 1A**). As shown in **Figure 1B through E**, similar results were observed for depression (low level of mindfulness: *B* = 0.52, *t* = 3.12, *p* < 0.01; high level of mindfulness: *B* = 0.42, *t* = 5.60, *p* < 0.01), anxiety (low level of mindfulness: *B* = 0.36, *t* = 1.38, *p* < 0.01; high level of mindfulness: *B* = 0.29, *t* = 2.54, *p* < 0.01), positive affect (low level of mindfulness: *B* = −0.19, *t* = −1.45, *p* < 0.01; high level of mindfulness: *B* = −0.39, *t* = −3.89, *p* < 0.01), and negative affect (low level of mindfulness: *B* = 0.47, *t* = 4.67, *p* < 0.01; high level of mindfulness: *B* = 0.33, *t* = 2.32, *p* < 0.05). While the overall mitigating effect of mindfulness on EE, depression, anxiety, and negative affect and the promoting effect of total mindfulness on positive emotion existed at both high and low levels of perceived stress, the effects were more significant when perceived stress levels were high.

**Figure 2:**
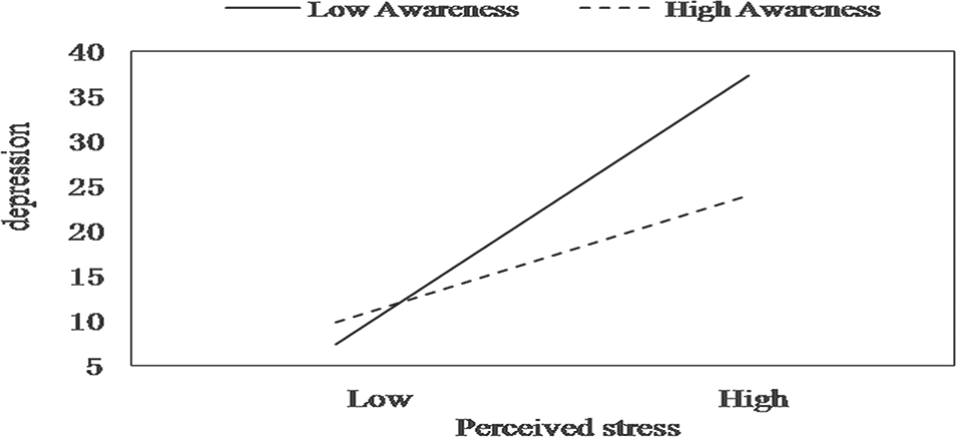
The moderating effect of acting with awareness on the relation between stress and depression.

After the entry of the five two-way interactions between perceived stress and mindfulness facets in step 4b, only one moderating effect was found. Acting with awareness moderated the relationship between perceived stress and depression. The simple slopes analysis indicated that ICU nurses with higher levels of acting with awareness had a weaker positive effect of perceived stress on depression (*B* = 0.71, *t* = 4.97, *p* < 0.01) than nurses with lower levels of acting with awareness (*B* = 0.34, *t* = 5.40, *p* < 0.01), and the mitigating effect of acting with awareness was pronounced when perceived stress levels were high (see **Figure 2**).

## Discussion

This study aimed to explore the potential moderating effect of mindfulness on the relationships among perceived stress and both positive and negative mental health indicators in Chinese intensive care nurses. This study found that the prevalence of psychological disorders such as burnout syndrome, anxiety, and depressive symptoms is common in Chinese ICU nurses, which is consistent with the previous studies (10, 18), and there was a higher prevalence of job-related burnout syndrome among ICU nurses than among general medical/surgical nurses (10). Although several studies have reported the effects of mindfulness on nurses (31, 60, 61), to our knowledge, no published studies have examined the moderating effect of mindfulness on stress-related mental health outcomes, especially burnout syndrome, in ICU nurses. After controlling for the effects of perceived stress and demographic variables of age and marital status, the present research confirmed that a higher level of mindfulness was associated with better psychological indicators in all outcome variables, except for personal achievement. These findings are in accordance with existing literature showing that higher levels of mindfulness may serve as a buffer for negative stress responses and consequently negative emotions such as burnout (28), depression (62, 63), and anxiety (62) and may also serve as a promoter for SWB (32, 64, 65).

The facet of observation could positively predict two positive indicators: life satisfaction and positive affect. Individuals who have a higher observation ability may be attuned to internal and external positive experiences and may be associated with the absence of ruminating thoughts during periods of high stress (35). Moreover, description was also significantly positively associated with two positive indicators, positive affect and personal achievement. Higher description ability may be important for effective communication and self-control (66), which may enable individuals to receive social support or facilitate successful task completion, thereby promoting positive affect and personal achievement. The facet of acting with awareness was significantly inversely correlated with all the negative indicators, including EE, DP, depression, anxiety, and negative affect. Several studies provide support for this prediction. Awareness is associated with a set of psychological benefits, such as reduced anxiety and depressive symptoms (24) and reduced EE (67). Using a single-item measure adapted from the facet of the acting with awareness subscale of the FFMQ (68), Donald et al. found that higher levels of awareness predicted greater perceived self-efficacy in dealing with stressful daily events, as increased awareness widens the range of available response options. Moreover, awareness exists independently of an individual’s level of perceived threat associated with the stressor and the degree of general negative affect the person experiences on a given day, thus facilitating more effective coping with daily stressors (68). Awareness was also proposed to improve an individual’s executive control (69), therefore mitigating the effect of perceived stress on mental health. Interestingly, in contrast to our predictions, non-judgment was negatively associated with personal achievement. Moreover, although non-reactivity to inner experience was positively correlated with positive affect, it was also positively correlated with DP. Only two studies were found to directly examine the interaction effects of facets of mindfulness and dimensions of burnout. Using a sample of 381 employees, Taylor et al. (70) found that all five facets of mindfulness were significantly and inversely correlated with the three components of burnout tested by the Maslach Burnout Inventory–General Survey (MBI-GS), with the exception of observation and EE. In a longitudinal cohort study of 27 clinicians who were offered a Mindfulness-Based Stress Reduction (MBSR) course, Dobkin et al. (71) found that the decrease in EE was correlated with acting with awareness and less-judgmental attitudes. Based on these results, we could posit that nonreactivity to inner experience refers to allowing thoughts and feelings to come and go without fixating on them, which may tend to detach ICU nurses’ responses to workplace stressors. Nonjudgment of inner experience refers to the ability to take a nonevaluative stance toward thoughts, which may reduce the motivation to pursue personal achievement. In their most recent research, Hafenbrack and Vohs (72) found that participants in the mindfulness group reported less motivation than did the participants in the comparison group. Another possible explanation for these findings is that different individuals interpret the meaning of the items in different ways, which might be due to different cultural values. As Maslach et al. (73) noted, the concept and measures of MBI had been established based on the culture in the United States. In China, sentiments of group solidarity play a more significant role, whereas individualism plays a major role in the North American society. Therefore, public expression of some dimensions of burnout syndrome, notably cynicism or DP, may be more widely acceptable in the United States than in China. As Rudkin et al. (74) found in their most recent reliable and valid research, the absence of certain items in the FFMQ may explain the novel function of some facets of FFMQ. The author then noted that their findings have implications for the development of multidimensional measures for mindfulness assessment. It has also been proposed that a bifactor structure might provide a better assessment than the existing five facets (75). Nevertheless, these novel findings may be interpreted to mean a number of things that are potentially important for the future research and understanding of mindfulness–burnout relations.

According to the results, the total level of mindfulness moderated the relationships among perceived stress and EE, depression, anxiety, positive affect, and negative affect. Feelings of EE are generally viewed as having the strongest correlation with the burnout variable (76); thus, EE is considered a core symptom of the burnout syndrome (77). The regression model results indicated that for ICU nurses with higher levels of mindfulness, the positive relationship between perceived stress and EE was weakened. However, for those with lower mindfulness levels, the relationship between perceived stress and EE was augmented. Therefore, we conclude that mindfulness may serve as a minimizer between perceived stress and EE. This finding is in accordance with a study conducted in Italy, which also suggested that dispositional mindfulness is important for protecting against the onset of burnout in healthcare professionals (78). Mindfulness might facilitate better self-regulation of emotional and cognitive activities and reduce reactions to potentially emotional and stressful stimuli (79). These improvements in appraising and reacting to potentially stressful events in ICU working environments help ICU nurses avoid EE (67).

Moreover, higher mindfulness levels are significantly correlated with lower perceived stress and lower rates of depression and anxiety. For ICU nurses with higher mindfulness levels, the positive relationship is weaker between perceived stress and depressive and anxiety symptoms. Within individuals with lower levels of mindfulness, this relationship is exactly the opposite. That is, the relationship between perceived stress and depressive and anxiety symptoms is weaker when levels of mindfulness are higher and vice versa. Therefore, mindfulness can be used as a buffer to reduce the tendency to experience more anxiety and depressive symptoms. Mindfulness moderated the relationship between perceived stress and depressive and anxiety symptoms in the sense that perceived stress was associated with fewer depressive and anxiety symptoms in ICU nurses with higher mindfulness scores. This is consistent with the results of many other studies including a large range of samples. In a group of law students, previous research found that total levels of mindfulness buffer the effects of perceived stress on depression and anxiety (35). A Swedish study tested the buffering role of mindfulness and found that the relationship between perceived stress and depression was attenuated for those with a higher level of mindfulness (34). Mindfulness has also been shown to improve anxiety and depression in cancer patients (80). Mindfulness, which focuses on being in the very present with a nonjudgmental and nonreactive mindset, may thereby alleviate the suffering that often accompanies depression and anxiety. This highlights the importance of taking a mindful approach toward internal cues to decrease depressive and anxiety symptoms.

Although mindfulness did not moderate the negative effects of stress on overall SWB or the component of life satisfaction, the results showed that mindfulness moderated the relationships of stress with positive affect and negative affect. These findings are particularly interesting, and somewhat ironic, as mindfulness is nonjudgmental, nonpreferential (neither toward or away from positive affect or negative affect), and nondisputational (81) but may, nevertheless, paradoxically strengthen the experience of positive affect or negative affect. An alternative explanation for our research result might be that the development of dispositional mindfulness may act as a buffer against stressors that result in reduced levels of positive affect across time and contexts (82). A very recent study also verified that mindfulness predicts greater improvements in positive affect and greater reductions in negative affect (83). ICU nurses who are naturally more mindful may have a decentered perspective of the mind or self and experience greater improvements in positive affect. Moreover, ICU nurses with higher, not lower, levels of mindfulness would be mindfully aware of and accept their internal experience, which may result in more benefits in light of negative affect reductions.

Further analyses focusing on particular mindfulness skills indicated that acting with awareness moderated the relationship between perceived stress and depression. This is the only facet of mindfulness buffering effect that emerged. This finding is consistent with the results of several other studies. By studying a sample of 520 Spanish adolescents and a subsample of 461 adolescents, Royuela-Colomer and Calvete (63) found that acting with awareness correlated negatively with depressive symptoms and predicted a reduction in depression over time. Pereira et al. (84) reported that acting with awareness had a protective effect against antenatal depressive symptoms in 427 pregnant women. In an exploratory factor analysis, Rudkin et al. (74) found that all meditator and nonmeditator participants identified the emotional awareness factor as the only factor that correlated with psychological symptoms. Caluyong et al. (85) also found that acting with awareness was a significant predictor of lower depression scores. This may be because the acting with awareness facet of mindfulness enables the detection of signs of stress and increases the ability of self-regulation and facilitates adaptive reactivity to negative and distressing situations. Thus, those with a higher ability to act with awareness have more potential to increase the awareness of low-level stress-related symptoms, which may potentiate access to coping resources and buffer against the negative effects of stress, such as depressive symptoms.

Based on these findings, interventions designed to encourage adaptive stress management, improve mindfulness levels, and provide the necessary skills to deal with stressful situations are likely to reduce or eliminate burnout syndromes and depressive and anxiety symptoms and improve the well-being of nurses working in the ICU. The results of the present study may help explain the relationships among perceived stress and mindfulness, burnout, anxiety, depression, and SWB. In addition, our results verified that mindfulness was a protective factor for alleviating or eliminating the negative effects of perceived stress on burnout, depression, anxiety, and SWB. In addition to its strengths, this study also has several limitations. First, we acknowledge that the data are based on ICU nurses’ self-reports. Current self-reported measurements of mindfulness may not accurately capture the constructs of the variables. A semistructured interview or longitudinal study may produce different results from those of the self-reported questionnaire used in our study. Second, as a cross-sectional study, this study failed to draw conclusions about the cause and effect relationships among the variables. For instance, whereas it seems that ICU nurses perceive less stress when they are more mindful, it might be possible that exposure to daily stressors results in decreased levels of mindfulness. Further longitudinal studies must be conducted.

## Conclusion

The high prevalence of prolonged stress, burnout syndromes, and depressive and anxiety symptoms in Chinese ICU nurses deserves immediate attention. The findings of the present study show that mindfulness is associated with lower levels of stress and fewer mental health complaints among Chinese ICU nurses. This study also indicates that mindfulness has the potential to act as a stress-coping resource. As a result, our findings suggest that mindfulness may be a target variable for health interventionists working with ICU nurses. Given the popularity of mindfulness, it might be a useful component of preventive health interventions and may allow for more targeted interventions.

## Ethics Statement

This study was carried out in accordance with the recommendations of the Ethics Committee of Army Medical University with written informed consent from all subjects. All subjects gave written informed consent in accordance with the Declaration of Helsinki. The protocol was approved by the Ethics Committee of Army Medical University.

## Author Contributions

FL, YX, and ML designed the research. FL, TWa, JX, BL, and SX recruited the participants and conducted the assessments. FL, YY, and TWu analyzed the data. FL wrote the manuscript, and YX and YY assisted with the statistical interpretations. YX, YY, LP, and TWa critically reviewed the manuscript. All of the authors contributed to the revision of the initial manuscript and approved the submission.

## Funding

This study was supported by the National Natural Science Foundation of China (no. 31700958), the Chongqing postgraduate innovation research foundation (no. CYB16120), the Science Research Foundation of Army Medical University (nos. 2016XRW09, 2016XYY06, and 2018XRW09), the Chongqing Technology Innovation and Application Demonstration Project (no. cstc2018jscx-msybX0119), the PLA Medical Innovation Project (no. 18CXZ005), and the National Social Science Foundation of China (no. 15CSH056).

## Conflict of Interest Statement

The authors declare that the research was conducted in the absence of any commercial or financial relationships that could be construed as a potential conflict of interest.
